# Liver Enzymes and Their Association with Some Cardiometabolic Diseases: Evidence from a Large Kurdish Cohort

**DOI:** 10.1155/2021/5584452

**Published:** 2021-06-11

**Authors:** Maryam Kohsari, Mehdi Moradinazar, Zohreh Rahimi, Yahya Pasdar, Ebrahim Shakiba

**Affiliations:** Behavioral Disease Research Center, Kermanshah University of Medical Sciences, Kermanshah, Iran

## Abstract

**Objective:**

According to reports, liver enzymes might play a role in the incidence and development of cardiometabolic diseases such as metabolic syndrome (MetS), hypertension (HTN), and cardiovascular diseases (CVD). We conducted a study to investigate this hypothesis among the Iranian Kurdish population.

**Methods:**

We analyzed data from the baseline phase of the Ravansar noncommunicable disease (RaNCD) cohort. The association between liver enzymes (ALT, AST, ALT/AST ratio, GGT, and ALP) with cardiometabolic disease risk factors was investigated by multiple linear regression. The odds ratio of cardiometabolic diseases in each quartile category of liver enzyme concentration was estimated using multivariable logistic regression.

**Results:**

The mean age of participants was 47.3 ± 4.1 years (48.1 years in males and 51.8 years in females). In the adjusted model, all enzymes were positively associated with MetS, HTN, and CVD risk factors except for the ALT/AST ratio with SBP and DBP. In the adjusted model, subjects in the fourth quartile for GGT, ALT/AST ratio, ALT, ALP, and AST had 3.29-, 2.94-, 2.45-, 2.00-, and 1.19-fold increased risk for MetS compared with subjects in the first quartile. Increased levels of GGT and ALP were positively associated with the risk of HTN (ORs = 1.33, 95%CI = 1.03–1.71 for GGT; ORs = 1.32, 95%CI = –1.68 for ALP). An increased GGT level was significantly associated with CVD (ORs = 1.54, 95%CI = 1.03–1.68). Within the normal range quartile, ALT had a significant correlation with the incidence of MetS.

**Conclusion:**

According to the present study, the levels of liver enzymes could be considered for early diagnosis of MetS, HTN, and CVD.

## 1. Introduction

Cardiometabolic diseases including cardiovascular disease (CVD), diabetes mellitus (DM), hypertension (HTN), metabolic syndrome (MetS), and chronic renal failure [1, 2] are the leading cause of noncommunicable disease (NCD) death in the world. Cardiometabolic diseases emerge as a result of an unhealthy lifestyle [2]. The risk factors for cardiometabolic diseases are abdominal obesity and elevated blood pressure, triglyceride, and fasting blood sugar and also decreased HDL-C [3]. Recently, the role of liver enzymes as a risk factor for cardiometabolic diseases has been considered [4–6]. The serum levels of the liver enzymes alanine aminotransferase (ALT), gamma-glutamyl transferase (GGT), aspartate aminotransferase (AST), and alkaline phosphatase (ALP) are markers of hepatocyte function [7] and elevated in various liver dysfunctions like nonalcoholic fatty liver disease (NAFLD) [4] as well as other metabolic disorders [8]. Studies on the population of China [4] and Korea [9] suggested that ALT and AST can be associated with MetS; also, a Korean cohort study demonstrated that GGT and aminotransferases were related to the development and mortality of CVD [6]. Rahman et al. demonstrated that elevated GGT and ALT levels correlated with the prevalence of HTN among Bangladeshi adults [5]. CVD is the first leading cause of mortality and DALYs in Iran [10], and prevalence rates of MetS [11] and HTN [12] are increasing (overall rates 42.3% for MetS and 25% for HTN). Finding new factors that help early diagnose cardiometabolic diseases can significantly reduce the mortality rate associated with these diseases.

Since there is no study to examine the relationship between liver enzymes and cardiometabolic diseases (MetS, HTN, and CVD) in the Kurdish population of Iran, this cross-sectional population-based study was conducted to assess the relationship between liver enzymes (ALT, AST, ALT/AST ratio, GGT, and ALP) and MetS, HTN, and CVD. We used the Ravansar noncommunicable disease (RaNCD) cohort's baseline data, which is the first longitudinal cohort study in a Kurdish settlement of the Middle East.

## 2. Material and Methods

### 2.1. Study Designed and Population

This study was conducted based on the data collected in the recruitment phase of the Ravansar noncommunicable disease (RaNCD) cohort. The RaNCD cohort is a part of the Prospective Epidemiological Research Studies of Iranian Adult (PERSIAN) cohort that focuses on permanent residents of Ravansar aged 35–65 years. In the PERSIAN cohort, all 19 cohort sites (covering a representative sample of different Iranian ethnicities) used the same questionnaire and are aimed at following up with all participants for the next 15 years. Further information is available at http://persiancohort.com and also in the cohort protocol [13, 14].

### 2.2. Inclusion and Exclusion Criteria

Inclusion criteria for the RaNCD cohort were willingness to participate and complete the research, being in the age range of 35-65, provision of a signed written informed consent letter, and ability to communicate with the research team. In this study, for elimination, the effect of confounding variables, participants with a history of renal failure, and pregnant women were excluded from the study. From 10,044 participants of the cohort, 9822 subjects were selected for this study.

### 2.3. Data Collection and Quality Control

Data were collected from face-to-face interviews by trained personnel at the cohort center. All selected participants received a reminder call the day before the appointment and were advised to fast. Demographic data such as lifestyle risk factors, medical history, and medication use for previous or current underlying diseases were obtained with a standardized questionnaire.

The blood samples were obtained with a venoject tube after overnight fasting. The serum was separated by centrifugation at 300 g for 10 minutes and stored at -20°C, until the testing day. Serum triacylglycerol (TG), high-density lipoprotein cholesterol (HDL-C), cholesterol, low-density lipoprotein (LDL), fasting blood glucose (FBG), and liver enzymes (including ALT, AST, GGT, and ALP) were analyzed with an enzymatic colorimetric assay by Mindray-BS-380 autoanalyzer (Mindray, USA).

### 2.4. Blood Pressure Measurement

Blood pressure was measured through a standardized procedure after 5 minutes of rest with two measurements of the right arm and two measurements of the left arm with cuff size adjusted to arm circumference. The cuff was placed on the arm at the heart level using a Riester duplex blood pressure device. There was at least a one-minute interval between two separate measurements. The average of two measurements for each arm was calculated. The higher measurement of two arms was considered the mean of systolic blood pressure (SBP) and diastolic blood pressure (DBP) [15].

This study considered participants with SBP ≥ 140 mm Hg and/or DBP ≥ 90 m Hg and/or those with current use of antihypertensive drugs as hypertensive individuals [15].

### 2.5. Smoking Status and Physical Activity

The smoking status of participants was evaluated based on the National Health Interview Survey (NHIS) [16]. 24-hour physical activity was measured by asking participants about their sport, work, and leisure-related activities on an average weekday based on METs/hour/day. MET is the amount of oxygen consumed at rest (about 3.5 ml O_2_/kg/min) and equals the resting metabolic rate. MET for each activity was extracted using a compendium of physical activities [17].

### 2.6. Anthropometric Measurements

We used a Bioimpedance Analyzer (BIA) (InBody 770 Biospace, Korea). Height was measured with 0.1 cm accuracy with a stadiometer. We calculated the participants' body mass index (BMI) by dividing weight (kg) by height (m^2^) [14]. Waist circumference (WC) was measured based on cm around the middle of the body at the upper part of the hip bones.

### 2.7. Metabolic Syndrome Criteria

Metabolic syndrome was specified according to the criteria published in 2009 [18]. The metabolic syndrome was identified by the presence of three or more of the following criteria: elevated TG ≥ 150 mg/dl, reduced HDL-C: <40 mg/dl in males and <50 mg/dl in females, and SBP ≥ 130 and DBP ≥ 86 mmHg or taking antihypertensive drugs defined as hypertension. Abdominal obesity was defined as elevated WC: ≥94 cm in males and ≥80 cm in females and elevated FBS ≥ 100 mg/dl.

### 2.8. Statistical Analysis

Categorical variables are expressed as the number and percent and evaluated by chi square. Continuous variables are examined by a *t*-test and reported as the mean and standard deviation (SD). In these tests, *p* value levels below 0.05 were considered significant. Simple and multiple linear regression was used to assess the relationship between liver enzymes and MetS, HTN, and CVD risk factors. The odds ratio (OR) of MetS, HTN, and CVD is according to quartiles of liver enzymes estimated with the multivariable logistic regression model. In regression models, data were presented as a *β* coefficient, odds ratio (OR), and 95% confidence intervals (CI). Statistical analyses were done with Stata software (version 14.2) (Stata Corp, College Station, TX, USA).

### 2.9. Ethical Considerations

The Ethics Committee of the Deputy of Research and Technology of Kermanshah University of Medical Sciences (KUMS.REC.1394.315) approved the study. All participants were well informed about the study process and entered the study after reading and signed the consent form.

## 3. Results

### 3.1. Baseline Characteristics of Study Subjects

Among the total of 9822 subjects, 48.1% were males and 51.8% were females. The prevalence of MetS, HTN, and CVD in the general population was 34.1%, 15.6%, and 16.6%, respectively.

The prevalence of MetS, HTN, and CVD in females was higher than in males. With increasing age, the incidence of cardiovascular diseases increased, so that the age range of 65-56 had the highest prevalence of MetS, HTN, and CVD among age groups. Compared with participants without cardiometabolic diseases, subjects with Mets, HTN, and CVD had higher WC, BMI, SBP, and DBP. The baseline characteristics of participants with and without cardiometabolic diseases are shown in Table 1.

### 3.2. Laboratory Test Baselines of Liver Enzymes and Other Risk Factors of Participants

Table 2 demonstrates the subjects with MetS having significantly higher levels of liver enzymes (ALT, AST, ALT/AST ratio, GGT, and ALP), TG, Chol, LDL, and FBS and lower level of HDL-C compared to those without MetS. HTN subjects had significantly higher levels of GGT, ALP, TG, LDL, Chol, and FBS than those with normal blood pressure, and CVD subjects compared with healthy individuals had a significantly lower ALT level and higher levels of GGT and ALP than the healthy group. The levels of Chol, TG, LDL, and FBS were higher in CVD subjects compared with healthy participants. Serum HDL levels in HTN and CVD were not significantly different from healthy individuals.

### 3.3. Association liver enzymes with other cardiometabolic risk factors

Table 3 shows the association between liver enzymes with MetS, HTN, and CVD risk factors by linear regression analysis. Results without the confounder effect showed that liver enzymes correlated with all risk factors except AST with WC. After adjusting the model, AST with FBS, ALT with WC, and ALT/AST ratio with SBP and DBP were not associated.

### 3.4. Association between Liver Enzymes and the Incidence Risk of Cardiometabolic Diseases

The ORs (with 95% CI) of MetS, HTN, and CVD across quartile categories of liver enzymes are presented in Table 4.

In the adjusted model, subjects in the fourth quartile for GGT, ALT/AST ratio, ALT, ALP, and AST had 3.29, 2.94, 2.45, 2.00, and 1.19-fold increased risk for MetS compared with subjects in the first quartile (ORs = 3.29, 95%CI = 2.70–4.02 for GGT; ORs = 2.94, 95%CI = 2.40–3.59 for ALT/AST; ORs = 2.45, 95%CI = 2.01–2.98 for ALT; ORs = 2.00, 95%CI = 1.66–2.41 for ALP; and ORs = 1.19, 95%CI = 1.00–1.43 for AST). The presence of fourth quartiles of GGT and ALP is associated with increased risk of HTN (ORs = 1.33, 95%CI = 1.03–1.71 for GGT; ORs = 1.32, 95%CI = 1.03–1.68 for ALP). Only an increased GGT level was significantly associated with CVD risk compared with first quartiles (ORs = 1.54, 95%CI = 1.03–1.68); also, increased ALP was associated with a CVD incidence but was not significant. Also, elevated levels of enzymes (ALT, AST, ALT/AST ratio, GGT, and ALP) gradually increased across quartiles and were associated with MetS prevalence. Also, elevated liver enzymes increased the HTN prevalence. The CVD prevalence with increasing levels of GGT and ALP enzymes within quartiles increased.

### 3.5. Association Incidence Risk of Cardiometabolic Diseases with Liver Enzymes in the Normal Range

Since only the normal range of ALT and AST enzymes in the Iranian population has been determined [19], we examined the incidence of MetS, HTN, and CVD in the normal range of these two enzymes.

Calculated ORs within the reference range are shown in Table 5. In the adjusted model, participants in the fourth quartile of ALT with 2.38-fold increased the risk of MetS (ORs = 2.38, 95%CI = 1.90–2.98). Although in the presence of a higher level of AST there was more likelihood incidence of MetS, it was not significant; also, there was a similar correlation between ALT and HTN incidence. The incidence of CVD increased within a normal range of ALT and AST, but it was not significant. However, the CVD prevalence did not increase within the quartiles.

## 4. Discussion

Cardiometabolic risk factors include abdominal obesity and elevated blood pressure, triglyceride, and fasting blood sugar and decreased HDL-C [3]; these factors together are often referred to as MetS; the presence of three or more of these pathophysiological conditions indicates having the disease in a person [18]. With rapid economic growth and urbanization, MetS prevalence has increased in the last three decades [20], so that in the study by Ansarimoghaddam et al. in 2014, overall MetS prevalence among the Iranian population has been reported to be 42.3% [11].

In our study among the Kurdish population from Western Iran, the prevalence of MetS was 33.3%, and the prevalence increased with age. MetS has also been associated with other clinical conditions such as cardiovascular disease [21], diabetes mellitus [22], hypogonadism, polycystic ovary syndrome, obstructive sleep apnea, vascular dementia, Alzheimer's disease, and carcinomas, especially pancreatic and colorectal cancers, hepatic steatosis, and nonalcoholic fatty liver disease (NAFLD) [23, 24]. In the field of NAFLD, some studies illustrated NAFLD as the liver manifestation of MetS; it seems that the context of each entity is overlapped. Most patients with NAFLD are asymptomatic [25]. Elevated ALT and AST levels are the first laboratory signs of NAFLD [26]; also, the ALT/AST ratio is commonly used as a sign of hepatic steatosis [27].

However, mechanisms underlying the association between NAFLD and cardiometabolic diseases had not been exactly elucidated yet [28]. In recent years, studies have focused on the liver enzymes' role in MetS and showed that liver enzymes might be novel candidate biomarkers for MetS [4, 9, 29, 30]. Wang et al. reported that high GGT and ALT levels were risk factors for MetS in Chinese [31]. Limited studies have been conducted among the Iranian population but not in the Kurdish ethnicity. Gaeini et al. in the Tehran Lipid and Glucose Study demonstrated that elevated serum concentrations of ALT, AST, ALT/AST ratio, GGT, and ALP were positively associated with an increased chance of developing MetS, and a liver function test could be used for the early detection of it [29].

The present study illustrated similar and parallel results. We documented a significant association of liver enzymes (ALT, AST, ALT/AST ratio, GGT, and ALP) with metabolic disorder factors of MetS without confounder effects and after adjustment for age, gender, smoking status, physical activity, and BMI. Besides, after dividing serum liver enzyme activity into quarterlies, it turns out that the increasing level of all included enzymes raised the prevalence and probability of MetS' occurrence. According to the theory, there is a connection between liver enzymes and metabolic syndrome within the normal range [32]. In two studies, Kim and Han in Korea [9] and Nikniaz et al. in East Azerbaijan, Iran, have studied this issue; the result showed that ALT and AST activities in high quartile were associated with a higher prevalence of MetS [30]. Due to the lack of any valid normal reference range for other liver enzymes in the Iranian population, we only included ALT and AST in our study [19]. Results demonstrated in the high quartile of ALT within the normal range that there was a higher prevalence of MetS and chance of MetS incidence. Although a high quartile of AST had a higher chance of MetS, it was not significant. Hypertension is related to impaired metabolic homeostasis and can be regarded as an independent metabolic disorder and a leading risk factor for CVD and premature death worldwide [33].

The incidence of HTN is increasing in developing countries like Iran. A meta-analysis study in 2019 indicated that the overall prevalence of HTN in Iranian society was 25%; that in men was slightly higher than in women [12]. HTN prevalence in our population was 15.5%, and females had a higher rate than males, and the elderly group had the highest prevalence of HTN. Numerous studies have shown an association between liver dysfunction and hypertension [5, 12, 34].

Almost 50% of HTN patients have NAFLD [35]. Sung et al. demonstrated that the development of a fatty liver was associated with an increased risk of hypertension [36]. A cross-sectional study by Rahman et al. indicated that serum ALT and GGT activities had an independent correlation with HTN and are elevated in hypertensive individuals [5]. The same result was obtained in the Park et al. study [37]. We found all studied activities of enzymes, except the ALT/AST ratio, strongly correlated with SBP and DBP. Further, there was an association between GGT and ALP with HTN; the highest quartiles of GGT and ALP were associated with the highest risk for HTN than the lowest quartile. We also assessed the possibility of high blood pressure in the normal range of ALT and AST. Results indicated that subjects in the fourth quartile compared to the first quartile had a higher risk for HTN that was not significant.

About 17 million people die each year from CVD [38]. According to the same pattern, CVD is the leading cause of daily death among the Iranian population [10]. In the capital city of Tehran, Iran, CVD approximately accounted for 40% of mortality [39]. The CVD prevalence had a relatively lower rate in our Kurdish population and was 16.6%. A meta-analysis study illustrated that GGT and ALP baseline in a log-linear was positively associated with CVD risk [40]. In the Korean cohort, the elevated serum levels of GGT, ALT, and AST were linked with the development and mortality of CVD [6]. Motamed et al., in a study consisting of 3199 subjects in Tehran, demonstrated that ALP was significantly associated with 10-year CVD risk in both men and women [39].

Our study showed that only an increased GGT level was significantly associated with CVD, and although increased ALP was associated with an incidence of CVD, it did not reach statistical significance. Also, Motamed et al. mentioned that ALT had a significant inverse association with the risk of CVD [39]. The results of the present study did not show this pattern, but subjects with CVD had a significantly lower serum level of ALT than healthy subjects. According to the results of this study, Gaeini et al. [29], and Motamed et al. [39], it seems that ALP, in particular, could be considered in the prognosis and prediction of cardiometabolic diseases in different Iranian populations. The clinical diagnosis of cardiometabolic diseases brings difficulty due to their complexity of pathogenesis and different manifestations. There is a necessity to find appropriate biomarkers for the early diagnosis of cardiometabolic diseases. The present study results indicated that the levels of liver enzymes could be helpful in laboratory diagnosis of cardiometabolic diseases.

### 4.1. Limitation and Strength

Due to financial constraints, we were not able to measure the assessed factors twice. For the first time in a Kurdish settlement in the Middle East, the present study assessed the association between liver enzymes and cardiometabolic diseases (including Mets, HTN, and CVD). To the best of our knowledge, this study is also the largest cross-sectional population-based study in Iran that examined this issue.

## 5. Conclusion

This study showed a significant association between elevated levels of ALT, AST, ALT/AST ratio, GGT, and ALP with increased risk of MetS. Liver enzyme levels are correlated with other risk factors for cardiometabolic diseases. The incidence of HTN is correlated with higher GGT and ALP levels, and only GGT was positively associated with CVD occurrence. Accordingly, based on the present study results, liver enzyme activity can help in the early diagnosis of cardiometabolic diseases (including MetS, HTN, and CVD).

## Figures and Tables

**Table 1:** Result obtained from recruitment phase according to metabolic syndrome, hypertension, and CVD.

Variables		Total	Metabolic syndrome		Hypertension		CVD	
			Yes	No	Yes	No	Yes	No
*N* (%)		9822 (100)	3265 (33.3)	6538 (66.7)	1526 (15.5)	8285 (84.6)	1632 (16.6)	8180 (83.4)
Gender, *N* (%)	Male	4730 (48.1)	1329 (40.7)	3397 (51.9)	683 (44.7)	4048 (48.9)^∗^	573 (35.1)	4158 (50.8)^∗^
	Female	5092 (51.8)	1936 (59.3)	3,141 (48.1)	843 (55.3)	4237 (51.1)	1059 (64.9)	4022 (49.2)^∗^

Age group (years)	35-45	4646 (47.3)	1153 (26.8)	3155 (73.2)^∗^	248 (5.4)	4378 (94.6)^∗^	315 (19.3)	4331 (52.9)
	46-55	3143 (32)	1135 (39)	1775 (61)	544 (17.4)	2584 (82.6)	561 (34.3)	2582 (15.6)
	56-65	2033 (20.7)	828 (43)	1099 (57)	712 (35.2)	1313 (64.8)	757 (46.4)	1276 (62.8)

Smoking status	Smoker	1145 (11.6)	361 (20.1)	701 (22.7)	125 (15.8)	1017 (23)^∗^	137 (16.5)	1008 (23.7)^∗^
	Nonsmoker	8677 (88.3)	1434 (79.9)	2384 (77.3)	665 (84.2)	3265 (77)	692 (83.5)	3234 (76.3)_∗_

Physical activity METs	Low	2891 (29.5)	841 (31.3)	1849 (68.7)	280 (9.7)	2603 (90.3)^∗^	357 (21.9)	2534 (31)^∗^
	Medium	4809 (49.2)	1507 (33.6)	2979 (66.4)	742 (15.5)	4045 (84.5)	788 (48.4)	4021 (49.3)
	High	2088 (21.3)	755 (39)	1182 (61)	480 (23.1)	1596 (76.9)	484 (29.7)	1604 (19.7)

Mean ± SD								
Waist circumference (cm)		97.2 ± 10.4	100.8 ± 9.3	95.2 ± 10.5^∗^	100.9 ± 10.6	96.6 ± 10.3^∗^	100.5 ± 10.5	96.6 ± 10.3^∗^
BMI (kg/m^2^)		27.4 ± 4.6	29.1 ± 4.2	26.6 ± 4.5^∗^	29 ± 4.7	27.2 ± 4.5^∗^	28.7 ± 4.6	27.2 ± 4.5^∗^
SBP (mmHg)		108.3 ± 16.9	116.8 ± 20.5	104.1 ± 12.9^∗^	130.2 ± 21	104.3 ± 12.5^∗^	119.5 ± 20.4	106.1 ± 15.2^∗^
DBP (mmHg)		69.9 ± 9.9	74.4 ± 11.7	67.7 ± 8^∗^	81.1 ± 11.8	67.8 ± 8^∗^	74.9 ± 11.3	68.8 ± 9.3^∗^

BMI: body mass index; SBP: systolic blood pressure; DBP: diastolic blood pressure. ^∗^*p* value < 0.001.

**Table 2:** Laboratory test baseline of liver enzymes and other risk factors of participants (mean ± SD).

Variables	Total	Metabolic syndrome		Hypertension		CVD	
		Yes	No	Yes	No	Yes	No
ALT (IU/l)	24.9 ± 14.6	27.6 ± 27.1	23.5 ± 13.9^∗∗^	24.4 ± 13	25 ± 14.9	24.1 ± 13.2	25 ± 14.9^∗^
AST (IU/l)	21.4 ± 8.8	21.8 ± 8.5	21.2 ± 8.9^∗∗^	21.3 ± 8.1	21.4 ± 8.9	21.2 ± 8.3	21.4 ± 8.9
ALT/AST ratio	1.1 ± 0.36	1.2 ± 0.37	1.0 ± 0.34^∗∗^	1.1 ± 0.34	1.1 ± 0.36	1.1 ± 0.34	1.1 ± 0.36
GGT (IU/l)	24.6 ± 19.9	28.9 ± 22.2	22.4 ± 18.2^∗∗^	26.8 ± 20.1	24.2 ± 19.6^∗∗^	26.6 ± 20	24.2 ± 19.8^∗∗^
ALP (IU/l)	197.9 ± 63.4	209.6 ± 73.6	191.7 ± 56.3^∗∗^	214.3 ± 89.7	194.8 ± 56.7^∗∗^	211 ± 86.7	195.2 ± 57.2^∗∗^
Chol (mg/dl)	185.4 ± 38	191.9 ± 38.8	181.7 ± 36.9^∗∗^	191.6 ± 40.3	184 ± 37.3^∗∗^	187.4 ± 40.4	184.7 ± 37.3^∗^
TG (mg/dl)	137.3 ± 81.7	198.2 ± 97.1	105.9 ± 48.7^∗∗^	153.9 ± 91.3	134.2 ± 79.4^∗∗^	149.7 ± 86.8	134.8 ± 80.5^∗∗^
LDL (mg/dl)	102 ± 25.4	106.2 ± 26.2	99.8 ± 24.9^∗∗^	105.7 ± 26.8	101.2 ± 25.1^∗∗^	103.2 ± 26.7	101.7 ± 25.1^∗^
HDL (mg/dl)	46.2 ± 11.3	40 ± 8.6	49.4 ± 11.1^∗∗^	46.1 ± 11	46.2 ± 11.3	46.1 ± 11.2	46.2 ± 11.3
FBS (mg/dl)	96.9 ± 29.7	109.2 ± 39.7	90.6 ± 20.2^∗∗^	106.8 ± 39.6	95.1 ± 27.2^∗∗^	107.7 ± 39.9	94.8 ± 26.7^∗∗^

ALT: alanine aminotransferase; AST: aspartate aminotransferase; GGT: gamma-glutamyl transferase; ALP: alkaline phosphatase; Chol: cholesterol; TG: triglyceride; LDL: low-density lipoprotein; HDL: high-density lipoprotein; FBS: fast blood glucose. ^∗^*p* value < 0.05. ^∗∗^*p* value < 0.001.

**Table 3:** Multivariate regression analysis of liver enzyme levels with cardiometabolic diseases.

Variables	ALT	AST	ALT/AST ratio	GGT	ALP
	*β* (95% CI)	*β* (95% CI)	*β* (95% CI)	*β* (95% CI)	*β* (95% CI)
Chol					
Crude	0.03 (0.02–0.04)	0.01 (0.01–0.02)	0.0004 (0.0002–0.0006)	0.07 (0.06–0.08)	0.23 (0.19–0.26)
Adjusted model	0.04 (0.03–0.05)	0.01 (0.01–0.02)	0.0008 (0.0006–0.001)	0.07 (0.05–0.08)	0.13 (0.09–0.18)

TG					
Crude	0.03 (0.02-0.03)	0.009 (0.007-0.01)	0.0009 (0.0008-0.001)	0.05 (0.04-0.05)	0.09 (0.08-0.11)
Adjusted model	0.02 (0.01–0.02)	0.005 (0.002-0.008)	0.0007 (0.0005-0.0008)	0.04 (0.03-0.05)	0.06 (0.04–0.08)

LDL					
Crude	0.05 (0.04–0.06)	0.02 (0.01–0.03)	0.0005 (0.0005–0.001)	0.1 (0.09–0.1)	0.28 (0.23–0.33)
Adjusted model	0.06 (0.04–0.08)	0.02 (0.01–0.03)	0.0009 (0.0005–0.001)	0.08 (0.06–0.1)	0.17 (0.10–0.24)

HDL					
Crude	-0.20 (-0.22–-0.17)	-0.02 (-0.0–-0.01)	-0.007 (-0.007–-0.006)	-0.16 (-0.20–-0.13)	-0.26 (-0.38–-0.10)
Adjusted model	-0.05 (-0.10–-0.01)	0.01 (-0.01–0.04)	-0.003 (-0.004–-0.002)	-0.06 (-0.12–-0.003)	-0.28 (-0.44–-0.10)

FBS					
Crude	0.04 (0.03–0.05)	-0.007 (-0.01–-0.001)	0.002 (0.002–0.002)	0.09 (0.08–0.11)	0.31 (0.27–0.36)
Adjusted model	0.04 (0.03–0.05)	-0.008 (-0.01–0.0001)	0.002 (0.002–0.002)	0.09 (0.07–0.11)	0.27 (0.21–0.32)

Waist circumference					
Crude	0.17 (0.14–0.19)	0.01 (-0.007–0.02)	0.007 (0.006–0.007)	0.22 (0.18–0.26)	0.47 (0.35–0.59)
Adjusted model	0.05 (-0.01–0.11)	0.01 (-0.02–0.05)	0.001 (0.0004–0.003)	0.09 (0.003–0.18)	0.37 (0.11–0.64)

SBP					
Crude	0.05 (0.04–0.07)	0.02 (0.01–0.03)	0.001 (0.0009–0.001)	0.11 (0.08–0.13)	0.52 (0.45–0.60)
Adjusted model	0.03 (0.006–0.05)	0.01 (0.001–0.03)	0.0002 (-0.0002–0.0008)	0.05 (0.01–0.08)	0.34 (0.24–0.45)

DBP					
Crude	0.10 (0.07–0.13)	0.04 (0.02–0.06)	0.002 (0.001–0.003)	0.18 (0.14–0.22)	0.75 (0.62–0.88)
Adjusted model	0.04 (0.08–0.08)	0.02 (-0.0002–0.05)	0.0002 (-0.0007–0.001)	0.09 (0.03–0.15)	0.51 (0.34–0.68)

ALT: alanine aminotransferase; AST: aspartate aminotransferase; GGT: gamma-glutamyl transferase; ALP: alkaline phosphatase; Chol: cholesterol; TG: triglyceride; LDL: low-density lipoprotein; HDL: high-density lipoprotein; FBS: fast blood glucose; SBP: systolic blood pressure; DBP: diastolic blood pressure. ^∗^Adjusted model for gender, age, smoking status, physical activity, and BMI.

**Table 4:** Odds ratio (OR) and 95% CI of the MetS, HTN, and CVD (according to quartiles of liver enzymes).

Variables	Prevalence^∗∗^ (%)	Metabolic syndrome	Prevalence^∗∗^ (%)	Hypertension	Prevalence^∗∗^ (%)	CVD
		Adjusted model^∗^ OR (95% CI)		Adjusted model^∗^ OR (95% CI)		Adjusted model OR (95% CI)
ALT						
Quartile 1	17.7	1.00	23.2	1.00	23.9	1.00
Quartile 2	22.1	1.17 (0.96–1.42)	26.9	0.92 (0.72–1.16)	27	0.95 (0.75–1.20)
Quartile 3	27	1.68 (1.38–2.03)	27.1	0.90 (0.70–1.15)	26.8	1.02 (0.80–1.29)
Quartile 4	33.2	2.45 (2.01–2.98)	22.8	0.91 (0.70–1.17)	22.3	1.008 (0.78–1.29)

AST						
Quartile 1	24.7	1.00	24.7	1.00	26	1.00
Quartile 2	23.7	0.95 (0.79–1.13)	24.9	0.94 (0.74–1.18)	25	0.97 (0.77–1.21)
Quartile 3	24.9	1.08 (0.91–1.30)	26.3	1.02 (0.81–1.29)	26	0.96 (0.76–1.21)
Quartile 4	26.7	1.19 (1–1.43)	24.1	1.02 (0.80–1.30)	23	1.02 (0.80–1.28)

ALT/AST ratio						
Quartile 1	15.9	1.00	24	1.00	24.3	1.00
Quartile 2	20.8	1.10 (0.89–1.35)	25.2	0.95 (0.74–1.22)	25.2	0.97 (0.76–1.23)
Quartile 3	28	1.78 (1.47–2.17)	26.8	1.00 (0.78–1.29)	26.5	0.98 (0.77–1.24)
Quartile 4	35.3	2.94 (2.40–3.59)	24	1.01 (0.78–1.30)	24	1.10 (0.85–1.41)

GGT						
Quartile 1	13.3	1.00	18.4	1.00	19.3	1.00
Quartile 2	21.1	1.65 (1.35–2.02)	23.6	1.03 (0.79–1.34)	23.3	1.07 (0.83–1.37)
Quartile 3	30.4	2.46 (2.01–3)	27.9	1.27 (0.99–1.64)	27	1.29 (1.01–1.66)
Quartile 4	35.2	3.29 (2.70–4.02)	30.1	1.33 (1.03–1.71)	30.3	1.54 (1.20–1.98)

ALP						
Quartile 1	18.7	1.00	19.1	1.00	19.6	1.00
Quartile 2	23.2	1.34 (1.10–1.62)	22.2	1.01 (0.78–1.31)	23.9	0.94 (0.73–1.21)
Quartile 3	26.3	1.52 (1.26–1.84)	23.5	0.87 (0.67–1.13)	25	0.82 (0.64–1.05)
Quartile 4	31.8	2.00 (1.66–2.41)	35.2	1.32 (1.03–1.68)	32.4	1.03 (0.81–1.31)

ALT: alanine aminotransferase; AST: aspartate aminotransferase; GGT: gamma-glutamyl transferase; ALP: alkaline phosphatase. ^∗^Adjusted model for gender, age, smoking status, physical activity, and BMI. ^∗∗^Without adjusted models.

**Table 5:** Odds ratio (OR) and 95% CI of the MetS, HTN, and CVD with the activity of the aminotransferases in the normal range.

Variables	Prevalence^∗∗^ (%)	Metabolic syndrome	Prevalence^∗∗^ (%)	Hypertension	Prevalence^∗∗^ (%)	CVD
		Adjusted model OR (95% CI)		Adjusted model OR (95% CI)		Adjusted model OR (95% CI)
ALT						
Quartile 1	17.7	1.00	23.2	1.00	23.9	1.00
Quartile 2	22.1	1.17 (0.96–1.42)	26.9	0.92 (0.72–1.16)	27	0.95 (0.75–1.20)
Quartile 3	27	1.68 (1.38–2.03)	27.5	0.90 (0.70–1.15)	26.8	1.02 (0.80–1.29)
Quartile 4	17.2	2.38 (1.90–2.98)	12.4	0.93 (0.69–1.26)	12.9	1.21 (0.90–1.61)

AST						
Quartile 1	24.7	1.00	24.7	1.00	26.2	1.00
Quartile 2	23.6	0.95 (0.79–1.13)	24.9	0.94 (0.74–1.28)	25.4	0.97 (0.77–1.21)
Quartile 3	24.9	1.08 (0.91–1.30)	26.7	1.02 (0.81–1.29)	25.3	0.96 (0.76–1.21)
Quartile 4	19.8	1.13 (0.93–1.38)	18.7	1.02 (0.79–1.33)	17.9	1.01 (0.72–1.50)

ALT: alanine aminotransferase; AST: aspartate aminotransferase. ^∗^Adjusted model for gender, age, smoking status, physical activity, and BMI. ^∗∗^Without adjusted models.

## Data Availability

The datasets used and/or analyzed during the current study are available from the corresponding author on reasonable request.

## References

[1] Whitesell P. L., Obi J., Tamanna N. S., Sumner A. E. (2018). A review of the literature regarding sleep and cardiometabolic disease in African descent populations. *Frontiers in Endocrinology*.

[2] de Waard A.-K. M., Hollander M., Korevaar J. C. (2019). Selective prevention of cardiometabolic diseases: activities and attitudes of general practitioners across Europe. *European Journal of Public Health*.

[3] Nichols G. A., Horberg M., Koebnick C. (2017). Cardiometabolic risk factors among 1.3 million adults with overweight or obesity, but not diabetes, in 10 geographically diverse regions of the United States, 2012–2013. *Preventing Chronic Disease*.

[4] Chen S., Guo X., Yu S., Zhou Y., Li Z., Sun Y. (2016). Metabolic syndrome and serum liver enzymes in the general Chinese population. *International Journal of Environmental Research and Public Health*.

[5] Rahman S., Islam S., Haque T., Kathak R. R., Ali N. (2020). Association between serum liver enzymes and hypertension: a cross-sectional study in Bangladeshi adults. *BMC Cardiovascular Disorders*.

[6] Choi K. M., Han K., Park S. (2018). Implication of liver enzymes on incident cardiovascular diseases and mortality: a nationwide population-based cohort study. *Scientific Reports*.

[7] Pratt D. S., Kaplan M. M. (2000). Evaluation of abnormal liver-enzyme results in asymptomatic patients. *New England Journal of Medicine.*.

[8] Sette L. H. B. C., Lopes E. P. (2014). Liver enzymes serum levels in patients with chronic kidney disease on hemodialysis: a comprehensive review. *Clinics*.

[9] Kim H. R., Han M. A. (2018). Association between serum liver enzymes and metabolic syndrome in Korean adults. *International Journal of Environmental Research and Public Health*.

[10] Sarrafzadegan N., Mohammmadifard N. (2019). Cardiovascular disease in Iran in the last 40 years: prevalence, mortality, morbidity, challenges and strategies for cardiovascular prevention. *Archives of Iranian Medicine*.

[11] Ansarimoghaddam A., Adineh H. A., Zareban I., Iranpour S., HosseinZadeh A., Kh F. (2018). Prevalence of metabolic syndrome in Middle-East countries: meta-analysis of cross-sectional studies. *Diabetes and Metabolic Syndrome: Clinical Research and Reviews*.

[12] Oori M. J., Mohammadi F., Norozi K., Fallahi-Khoshknab M., Ebadi A., Gheshlagh R. G. (2019). Prevalence of HTN in Iran: meta-analysis of published studies in 2004-2018. *Current Hypertension Reviews*.

[13] Poustchi H., Eghtesad S., Kamangar F. (2018). Prospective epidemiological research studies in Iran (the PERSIAN Cohort Study): rationale, objectives, and design. *American journal of epidemiology.*.

[14] Pasdar Y., Najafi F., Moradinazar M. (2019). Cohort profile: Ravansar Non-Communicable Disease cohort study: the first cohort study in a Kurdish population. *International Journal of Epidemiology*.

[15] Chobanian A. V., Bakris G. L., Black H. R. (2003). The seventh report of the joint national committee on prevention, detection, evaluation, and treatment of high blood pressure: the JNC 7 report. *Journal of the American Medical Association*.

[16] Ryan H., Trosclair A., Gfroerer J. (2012). Adult current smoking: differences in definitions and prevalence estimates—NHIS and NSDUH, 2008. *International Journal of Environmental Research and Public Health*.

[17] Aadahl M., Jørgensen T. (2003). Validation of a new self-report instrument for measuring physical activity. *Medicine and Science in Sports and Exercise*.

[18] Alberti K., Eckel R. H., Grundy S. M. (2009). Harmonizing the metabolic syndrome: a joint interim statement of the International Diabetes Federation Task Force on Epidemiology and Prevention; National Heart, Lung, and Blood Institute; American Heart Association; World Heart Federation; International Atherosclerosis Society; and International Association for the Study of Obesity. *Circulation*.

[19] Mohamadnejad M., Pourshams A., Malekzadeh R. (2003). Healthy ranges of serum alanine aminotransferase levels in Iranian blood donors. *World Journal of Gastroenterology*.

[20] Saklayen M. G. (2018). The global epidemic of the metabolic syndrome. *Current Hypertension Reports*.

[21] Higashiyama A., Okamura T., Ono Y., Watanabe M., Kokubo Y., Okayama A. (2009). Risk of smoking and metabolic syndrome for incidence of cardiovascular disease Comparison of Relative Contribution in Urban Japanese Population: The Suita Study. *Circulation*.

[22] Lorenzo C., Okoloise M., Williams K., Stern M. P., Haffner S. M., San Antonio Heart Study (2003). The metabolic syndrome as predictor of type 2 diabetes: the San Antonio heart study. *Diabetes Care*.

[23] Grundy S. M. (2008). Metabolic syndrome pandemic. *Arteriosclerosis, Thrombosis, and Vascular Biology*.

[24] Uzunlulu M., Telci Caklili O., Oguz A. (2016). Association between metabolic syndrome and cancer. *Annals of Nutrition & Metabolism*.

[25] Hamaguchi M., Kojima T., Takeda N. (2005). The metabolic syndrome as a predictor of nonalcoholic fatty liver disease. *Annals of Internal Medicine*.

[26] Angulo P., Keach J. C., Batts K. P., Lindor K. D. (1999). Independent predictors of liver fibrosis in patients with nonalcoholic steatohepatitis. *Hepatology*.

[27] Kain K., Carter A. M., Grant P. J., Scott E. M. (2008). Alanine aminotransferase is associated with atherothrombotic risk factors in a British South Asian population. *Journal of Thrombosis and Haemostasis*.

[28] El Hadi H., Di Vincenzo A., Vettor R., Rossato M. (2019). Cardio-metabolic disorders in non-alcoholic fatty liver disease. *International Journal of Molecular Sciences*.

[29] Gaeini Z., Bahadoran Z., Mirmiran P., Azizi F. (2020). The association between liver function tests and some metabolic outcomes: Tehran Lipid and Glucose Study. *Hepatitis Monthly*.

[30] Nikniaz L., Nikniaz Z., Tabrizi J. S., Sadeghi-Bazargani H., Farahbakhsh M. (2018). Is within-normal range liver enzymes associated with metabolic syndrome in adults?. *Clinics and Research in Hepatology and Gastroenterology*.

[31] Wang S., Zhang J., Zhu L. (2017). Association between liver function and metabolic syndrome in Chinese men and women. *Scientific Reports*.

[32] Marceau P., Biron S., Hould F.-S. (1999). Liver pathology and the metabolic syndrome X in severe obesity. *The Journal of Clinical Endocrinology and Metabolism*.

[33] Mills K. T., Stefanescu A., He J. (2020). The global epidemiology of hypertension. *Nature Reviews. Nephrology*.

[34] Gupta A., Panthari M., Ahmad N., Nagtilak S., Nandwani S. (2013). Levels of alanine aminotransferase (ALT), aspartate amino transferase (AST) and gamma glutamyl transferase (GGT) in hypertension. *Biomedical Research*.

[35] Donati G., Stagni B., Piscaglia F. (2004). Increased prevalence of fatty liver in arterial hypertensive patients with normal liver enzymes: role of insulin resistance. *Gut*.

[36] Sung K.-C., Wild S. H., Byrne C. D. (2014). Development of new fatty liver, or resolution of existing fatty liver, over five years of follow-up, and risk of incident hypertension. *Journal of Hepatology*.

[37] Park E.-O., Bae E. J., Park B.-H., Chae S.-W. (2020). The associations between liver enzymes and cardiovascular risk factors in adults with mild dyslipidemia. *Journal of Clinical Medicine*.

[38] W WHO (2013). *A global brief on hypertension: silent killer, global public health crisis*.

[39] Motamed N., Rabiee B., Farahani B. (2016). Association of liver enzymes with 10-year cardiovascular disease risk: a population-based study. *Hepatitis Monthly*.

[40] Kunutsor S. K., Apekey T. A., Khan H. (2014). Liver enzymes and risk of cardiovascular disease in the general population: a meta-analysis of prospective cohort studies. *Atherosclerosis*.

